# Supplementary material for: Investigating the Acceptability of Cervical Screening and Self‐Sampling in Postnatal Women at the 6‐Week Postnatal Check‐Up: A Qualitative Study
Source: Health Expect. 2026 Feb 4;29(1):e70582. doi: 10.1111/hex.70582 (PMC12873452; doi:10.1111/hex.70582)
Supplement: Supplementary file 3 — Supplementary Table 1. Participant characteristics. [file HEX-29-e70582-s003.docx]

| **Participant number** | **Screening history** | **When did give birth (how many children)** | **Willing to take part in study with cervical screen at 6 weeks?** | **Willing to take part in study cervical screen if urine sample 6 weeks** | **More like to have smear after pregnancy if at 6-week check** | **More likely to have cervical screening 6 weeks if only urine sample** | **Would prefer to do self-testing with urine sample than smear** | **Prefer cervical screening 6 weeks than 12 weeks** | **Ethnicity** | **Age** | **Employment** | **Highest education** |
| --- | --- | --- | --- | --- | --- | --- | --- | --- | --- | --- | --- | --- |
| 16 | Over 3yrs | Within last 4m (1 child) | Yes | Yes | Disagree | Strongly agree | Strongly agree | Disagree | E, W, S, NI or Bri | 24-29 | Employed/self-employed | College/University |
| 25 | Over 3 yrs | 3-4yrs ago  (1 child) | Yes | Yes | Agree | Disagree | Disagree | Neutral | E, W, S, NI or Bri | 35-39 | Employed/self-employed | College/University |
| 30 | Within last 3 yrs | Within last 4m  (2 children) | Yes | Yes | Neutral | Neutral | Strongly disagree | Strongly agree | E, W, S, NI or Bri | 30-34 | Employed/self-employed | Higher/secondary/further education (A Levels) |
| 45 | Over 3 yrs | Within last 4m  (1 child) | Yes | Yes | Strongly agree | Strongly agree | Neutral | Agree | E, W, S, NI or Bri | 24-29 | Employed/self-employed | College/University |
| 49 | Within past 3yrs | 2-3yrs ago  (1 child) | Yes | Yes | Neutral | Neutral | Agree | Neutral | E, W, S, NI or Bri | 30-34 | Employed/self-employed | Post-graduate |
| 51 | Over 3yrs | Within past 4m  (3 children) | Yes | Yes | Strongly agree | Strongly agree | Strongly agree | Strongly agree | E, W, S, NI or Bri | 35-39 | Employed/self-employed | Post-graduate |
| 71 | Never (24) | Within past 4m  (2 children) | Yes | Yes | Strongly agree | Strongly agree | Strongly agree | Agree | E, W, S, NI or Bri | 24-29 | Homemaker | Higher/secondary/further education (A Levels) |
| 93 | Over 3 yrs | 4-12m ago  (2 children) | Yes | Yes | Strongly agree | Neutral | Strongly disagree | Neutral | E, W, S, NI or Bri | 30-34 | Homemaker | College/university |
| 115 | Within last 3 yrs | Within last 4m (1 child) | No | Yes | Disagree | Strongly agree | Strongly agree | Strongly disagree | E, W, S, NI or Bri | 24-29 | Employed/self employed | Post-graduate |
| 120 | Within last 3 yrs | Within last 4m (1 child) | Yes | Yes | Agree | Neutral | Disagree | Agree | E, W, S, NI or Bri | 24-29 | Employed/self-employed | Post-graduate |
| 153 | Within last 3 yrs | Pregnant  (0 child) | Yes | Yes | Agree | Strongly agree | Agree | Agree | E, W, S, NI or Bri | 30-34 | Employed/self-employed | Post-graduate |
| 161 | Within past 3yrs | Within past 4m  (2 children) | Yes | Yes | Strongly agree | Agree | Agree | Strongly agree | E, W, S, NI or Bri | 30-34 | Employed/self-employed | Post-graduate |
| 185 | Within past 3yrs | Within last 4m  (3 children) | Yes | Yes | Strongly agree | Agree | Neutral | Strongly agree | E, W, S, NI or Bri | 30-34 | Employed/self employed | Higher/secondary/further education (A Levels) |
| 186 | Within last 3 yrs | 4-12m ago  (3 children) | Yes | Yes | Neutral | Neutral | Disagree | Neutral | E, W, S, NI or Bri | 30-34 | Employed/self-employed | College/University |
| 202 | Within last 3 yrs | Within last 4m (1 child) | No | Yes | Strongly disagree | Strongly disagree | Strongly agree | Strongly disagree | E, W, S, NI or Bri | 24-29 | Employed/self-employed | Post-graduate |
| 212 | Within last 3 yrs | 2-3 yrs ago  (1 child) | No | Yes | Neutral | Agree | Agree | Neutral | E, W, S, NI or Bri | 24-29 | Employed/self-employed | Post-graduate |
| 216 | Over 3 yrs | Pregnant  (1 child) | No | Yes | Neutral | Strongly agree | Strongly agree | Neutral | E, W, S, NI or Bri | 40-44 | Homemaker | College/University |
| 240 | Within last 3 yrs | Within last 4m (1 child) | Yes | Yes | Strongly agree | Neutral | Disagree | Strongly agree | E, W, S, NI or Bri | 30-34 | Employed/self-employed | Post-graduate |
| 241 | Within last 3 yrs | 4-12m ago  (2 children) | Yes | Yes | Strongly agree | Strongly agree | Neutral | Strongly agree | E, W, S, NI or Bri | 24-29 | Employed/self-employed | College/University |
| 281 | Within last 3 yrs | Within last 4m  (2 children) | Yes | Yes | Strongly agree | Strongly agree | Neutral | Strongly agree | E, W, S, NI or Bri | 24-29 | Employed/self-employed | Post-graduate |
| 289 | Within last 3 yrs | Within last 4m (1 child) | Yes | Yes | Strongly agree | Strongly agree | Strongly agree | Neutral | E, W, S, NI or Bri | 30-34 | Employed/self employed | College/University |
| 302 | Over 3 yrs | Pregnant  (1 child) | Yes | Yes | Agree | Neutral | Agree | Neutral | E, W, S, NI or Bri | 35-39 | Employed/self-employed | College/University |
| 348 | Over 3 yrs | Within last 4m  (3 children) | Yes | Yes | Strongly agree | Strongly agree | Strongly agree | Strongly agree | E, W, S, NI or Bri | 35-39 | Unemployed | Higher/secondary/further education (A Levels) |
| 362 | Within last 3 yrs | 4-12m ago  (1 child) | Yes | Yes | Strongly agree | Strongly agree | Strongly agree | Agree | E, W, S, NI or Bri | 30-34 | Employed/self-employed | College/University |
| 365 | Over 3yrs | 4-12m ago  (1 child) | Yes | Yes | Disagree | Disagree | Agree | Agree | Any other ethnic group | 30-34 | Employed/self-employed | Post-graduate |
| 385 | Over 3yrs | Within past 4m (1 child) | Yes | Yes | Disagree | Strongly agree | Strongly agree | Disagree | E, W, S, NI or Bri | 24-29 | Employed/self-employed | Post-graduate |

**Supplementary Table 1. Participant characteristics**

Number of participants = 26. E, W, S, NI or Bri = White English, Welsh, Scottish, Northern Irish or British origin; m = months; yrs = years
